# Olaparib Enhances the Efficacy of Third‐Generation Oncolytic Adenoviruses Against Glioblastoma by Modulating DNA Damage Response and p66shc‐Induced Apoptosis

**DOI:** 10.1111/cns.70124

**Published:** 2024-11-18

**Authors:** Yida Liu, Sheng Fang, Peiwen Wang, Junwen Zhang, Fusheng Liu

**Affiliations:** ^1^ Brain Tumor Research Center, Beijing Neurosurgical Institute Capital Medical University Beijing China; ^2^ Department of Neurosurgery Beijing Tiantan Hospital Affiliated to Capital Medical University Beijing China; ^3^ Beijing Laboratory of Biomedical Materials Beijing China

**Keywords:** apoptosis, DNA damage, glioblastoma, oncolytic adenovirus, p66shc, PARP

## Abstract

**Aims:**

Patients with glioblastoma multiforme (GBM) do not benefit from current cancer treatments, and their prognosis is dismal. This study aimed to investigate the potential synergistic effects of TS‐2021, a third‐generation oncolytic adenovirus, combined with the PARP inhibitor olaparib in GBM.

**Methods:**

TS‐2021’s impact on p66shc‐induced apoptosis, DNA damage response, and poly (ADP‐ribose) polymerase (PARP) activation was evaluated in GBM cells. The synergistic effect of TS‐2021 and olaparib was examined in GBM cell lines and an immunocompetent mouse model of GBM. Mechanistic studies focused on the role of p66shc phosphorylation in the observed effects.

**Results:**

TS‐2021 triggered p66shc‐induced apoptosis, DNA damage response, and PARP activation. The combination of TS‐2021 and olaparib synergistically increased cell apoptosis and DNA damage and reduced PARP expression compared to monotherapy. Olaparib promoted TS‐2021 replication and release in GBM cells. Mechanistically, olaparib combined with TS‐2021 upregulated p66shc phosphorylation, enhancing tumor cell apoptosis. In vivo, the combination therapy inhibited tumor growth and prolonged survival, confirming the synergistic effect.

**Conclusion:**

This study is the first to suggest that TS‐2021 sensitizes GBM cells with wild‐type BRCA1/2 to olaparib. The combination of TS‐2021 and olaparib shows a synergistic therapeutic effect against GBM, providing a promising treatment strategy.

## Graphical abstract

On the basis of oncolytic adenovirus TS‐2021 infection caused PARP activation. The combination of TS‐2021 and olaparib increases virus titer, p66shc phosphorylation, reactive oxygen species production, and DNA damage, leading to synergistic apoptosis in GBM.

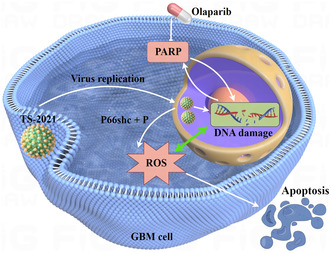

## 1 Introduction

Glioblastoma multiforme (GBM) is the most common primary intracranial malignant tumor with a poor prognosis [1, 2]. The current main treatment methods include surgery, radiochemotherapy, tumor treating field therapy, targeted therapy, and immunotherapy [3]. With the continuous advancement of gene detection technology and the introduction of new concepts in immunotherapy, targeted therapy and immunotherapy have emerged as new hopes in the field of GBM treatment [4, 5]. However, both the development of targeted drugs and research on immunotherapy still lack mature and reliable clinical data to validate their efficacy.

Oncolytic virotherapy, a novel immunotherapy, utilizes replication‐ability viruses to trigger host antitumor immunity, target tumor cells, and cause immunogenic cell death, offering a promising and safe treatment for GBM [6, 7]. Since 2018, our team has developed a new generation of genetically engineered oncolytic herpes simplex virus and oncolytic adenovirus (oAd). Previously, we have shown that oncolytic virotherapy can exert antitumor efficacy by affecting tumor cell metabolism, suppressing invasion by downregulating Sp1, attenuating PD‐L1 expression, and activating macrophage polarization [8, 9, 10, 11]. TS‐2021, a third‐generation oAd that carried Ki67 promoter, transforming growth factor‐β2 (TGF‐β2) 5′UTR and IL‐15, has shown promising therapeutic effects in clinical trials for treating recurrent GBM. TS‐2021 replication is driven by the ki67 promoter and the TGF‐β2 5′UTR, directly associated with tumor malignancy, providing greater targeting specificity. Additionally, it can regulate the replication and release of downstream IL‐15, further activating immune cells to trigger an anti‐tumor response [12]. However, oAd, as a DNA virus, can trigger DNA damage response (DDR) in infected cells [13], eliciting host antiviral response to limit viral infection by identifying viral genomes and virus‐induced DNA damage [14]. Combining TS‐2021 with a therapeutic agent that offsets the host antiviral response would enhance its efficacy, improving clinical outcomes.

Poly (ADP‐ribose) polymerase (PARP) inhibitors block DDR to increase DNA damage accumulation and induce cell apoptosis, especially in homologous recombination (HR)‐deficient cells, such as *BRCA* mutant cells [15]. Although monotherapy with PARP inhibitors is not satisfactorily efficacious for GBM cells with wild‐type *BRCA1/2* [16, 17], DNA‐damaging agents such as temozolomide (TMZ) and radiation increase GBM sensitivity to the PARP inhibitor olaparib [18, 19]. In other words, DNA damage can enhance olaparib‐mediated killing of tumor cells. Importantly, olaparib has been approved by the United States Food and Drug Administration for use as a maintenance drug after first‐line chemotherapy for ovarian cancer [20]. Therefore, combining the potent DNA‐damaging agent TS‐2021 with olaparib may prove beneficial for treating GBM, and further research into its potential mechanisms could aid in improving patient prognosis.

We first observed that TS‐2021 infection caused PARP activation. In this study, we further investigated the combined effect of TS‐2021 and olaparib on GBM. Combination therapy increased virus titer, p66shc phosphorylation, reactive oxygen species (ROS) production, and DNA damage, leading to synergistic apoptosis in GBM cells and improved antitumor effect in vivo and in vitro. Furthermore, we proposed a novel treatment strategy for GBM for the first time, combining the oAd TS‐2021 with the PARP inhibitor olaparib, and explored the potential mechanisms underlying the effects of this combination therapy.

## 2 Materials and Methods

### 2.1 Cell Lines and Reagents

The GBM cell lines U251 and U87 were purchased from the Cell Center, Chinese Academy of Medical Science (Beijing, China) and tested for mycoplasma contamination with a Mycoplasma Detection Kit (Yeasen, Shanghai, China). As described previously, we established BT‐01 and U87‐Luc cell lines [11, 21]. All cells were cultured in Dulbecco's Modified Eagle Medium (Gibco, USA) containing 10% fetal bovine serum (Gibco, USA) and 100 U/mL penicillin/streptomycin (Gibco, USA) in an incubator at 37°C with 5% CO_2_ and pure water. Olaparib (MedChemExpress, USA) was dissolved in dimethyl sulfoxide to a final concentration of 1 mM for in vitro studies. For in vivo studies, olaparib was solubilized in dimethyl sulfoxide and diluted to 2.5 mg/mL with 20% sulfobutylether‐β‐cyclodextrin (Yuanye Bio‐technology, Shanghai, China) in saline.

### 2.2 Oncolytic Adenovirus

The oAds TS‐2021/Ad5 KT‐E1A‐IL15 and AdGFP/Ad5 KT‐E1A‐GFP have been described in our previous report [12].

### 2.3 Transmission Electron Microscopy

U251, U87, and BT‐01 cells were cultured with TS‐2021 at a multiplicity of infection (MOI) of 300 for 72 h. Thereafter, the cells were washed with phosphate‐buffered saline (PBS) and fixed in osmium tetroxide. After dehydrating in graded alcohol, the cells were sectioned into thin slices and stained with alkaline lead citrate and uranyl acetate. Finally, images were captured by transmission electron microscopy (Hitachi, Japan).

### 2.4 Flow Cytometry

#### 2.4.1 Cell Cycle Analysis

Cells were harvested, washed in PBS, fixed in 70% alcohol, and incubated at −20°C for 1 h after different treatments. The samples were washed with PBS and then incubated with 50 μL RNase A (100 mg/mL) in a water bath at 37°C for 30 min. The samples were then mixed with 200 μL of propidium iodide (PI, 50 μg/mL) and kept in the dark at 4°C for another 30 min. After collection, the samples were analyzed using a flow cytometer.

#### 2.4.2 Apoptosis Analysis

Cells were collected after different treatments and washed once with PBS, then double‐stained using the Annexin V‐FITC/PI Apoptosis Kit (Elabscience, Hubei, China) according to the manufacturer's instructions. After collection, the samples were assessed using a flow cytometer.

#### 2.4.3 ROS‐Generation Analysis

Cells harvested after different treatments were mixed with dichlorodihydrofluorescein diacetate (DCFH‐DA, 10 μM/L) at 37°C for 1 h using the Reactive Oxygen Species Assay Kit (Beyotime, Shanghai, China). Real‐time fluorescence intensities of the samples were detected at an excitation wavelength of 488 nm and an emission wavelength of 525 nm.

All samples were assessed using Accuri C6 flow cytometers (BD biosciences, USA) within 1 h of preparation, and the data were calculated using FlowJo (Tree Star, USA).

### 2.5 Immunofluorescence Assay

U251, U87, and BT‐01 cell lines were seeded in six‐well plates (2 × 10^5^ cells/well) with indicated treatment for 72 h. Following the manufacturer's instructions, the cells were double‐stained using the DNA Damage Assay Kit (Beyotime, Shanghai, China). Phosphorylated H2AX (γH2AX) expression was observed with a fluorescence microscope (ZEISS, Germany), and the data were analyzed using ImageJ (NIH, USA).

### 2.6 Western Blotting

The extracted cell proteins were separated by sodium dodecyl sulfate‐polyacrylamide gel electrophoresis and transferred onto nitrocellulose filter membranes (Millipore, Billerica, MA), which were then blocked in blocking buffer (Beyotime, Shanghai, China) and incubated with the following primary antibodies: anti‐caspase‐3 (1:1000, 9665; CST), anti‐PARP (1:1000, 9532; CST), anti‐γH2AX Ser139 (1:1000, AF5836; Beyotime, Shanghai, China), anti‐PAR (1:1000, 83732; CST), anti‐p66shc (1:1000, A7725; ABclonal, Hubei, China), and anti‐phospho‐Ser36‐p66shc (1:1000, YP1075; Immunoway, USA). The membranes were then incubated with a secondary antibody (1:2000, S0100, S0101; LABLEAD, Beijing, China) after washed with PBS‐tween‐20. The blots were developed using an ECL Western Blotting Detection Kit (Invitrogen, USA). A monoclonal β‐actin antibody (1:5000; LABLEAD, Beijing, China) was used to analyze protein loading.

### 2.7 Evaluation of Viral Infectivity, Replication, and Release

The three GBM cell lines were seeded in six‐well plates at a density of 10^5^ cells per well. The cells were then treated with olaparib (1 μM) and infected with either AdGFP (MOI = 300). Green fluorescent protein (GFP) fluorescence intensity at 48 h post‐infection was assessed using a fluorescence microscope. The average fluorescence intensity of a single cell was determined using Image J.

Cells and supernatant were separately collected 48 h after desired treatment. Extract DNA from cells and supernatant separately using the TIANamp Genomic DNA Kit (Tiangen Biotech, Beijing, China). As previously described [22], quantitative polymerase chain reaction (qPCR) was performed to evaluate viral replication and release. A standard reference curve (Figure S1) was plotted using TS‐2021 (5.0 × 10^11^ vp/mL). The *E1A* primer sequences used were as follows:

– 5′‐AACCAGTTGCCGTGAGAGTTG‐3′ (forward),
– 5′‐TCGTTAAGCAAGTCCTCCTCGATACAT‐3′ (reverse).

### 2.8 Small Interfering RNA Transfection

Cells were transfected with p66shc small interfering RNA (siRNA) or control siRNA (RiboBio, Guangdong, China), following the supplier's protocol. The expression of p66shc 48 h post‐transfection was analyzed by western blotting. The siRNA sequences were as follows:

– 5′‐GCTACCACATGGACAATCA‐3′ (siRNA1#),
– 5′‐CCAGAAGTCCGCAAACAGA‐3′ (siRNA2#),
– 5′‐GAGTTGCGCTTCAAACAAT‐3′ (siRNA3#).

### 2.9 Animal Experiments

All animal experiments were approved by the Experimental Animal Ethics Committee of the Beijing Neurosurgical Institute (BNI202307009). Female BALB/c nude mice (age, 6 weeks) were obtained from Gempharmatech Co. Ltd. (Jiangsu, China) and kept in a specific pathogen‐free environment. Orthotopic tumor models were established following the procedure described earlier [11]. First, mice were anesthetized using isoflurane. Next, 2 × 10^5^ U87‐Luc cells were injected into the right striatum, located 2 mm lateral and 1 mm anterior to the bregma, at a depth of 3 mm from the brain surface.

Seven days post‐injection, mice were imaged with an IVIS instrument (IVIS Spectrum, USA) to evaluate tumor growth. Tumor‐bearing mice were divided randomly into four treatment groups (10 mice/group): control, olaparib, TS‐2021, and combination groups. Olaparib (50 mg/kg body weight) was administered via oral gavage for five consecutive days a week. TS‐2021 (8 μL, 5.0 × 10^11^ vp/mL) was injected in situ once a week.

Tumor growth image data in vivo were acquired from the IVIS Spectrum on days 7, 14, 21, and 28 after the orthotopic implantation. Mouse weight and survival time were determined every 7 days post‐treatment. At the endpoint, we collected heart, liver, spleen, lung, and kidney specimens from three mice in each group. The tissues were fixed with 4% paraformaldehyde and embedded in paraffin. Tissue sections embedded in paraffin were stained with hematoxylin and eosin for visualization.

### 2.10 Statistical Analyses

All results were reported as mean ± standard deviation. A *p* value < 0.05 was considered significant. Statistical analyses were performed using *t*‐tests or one‐way ANOVA with Tukey's multiple comparisons tests. The survival percentage was calculated using the Kaplan–Meier method and log‐rank test. All analyses were conducted using GraphPad Prism 9.4 (Domatics, UK).

## 3 Results

### 3.1 TS‐2021 Infection Induced Apoptosis in GBM Cells

**FIGURE 1:**
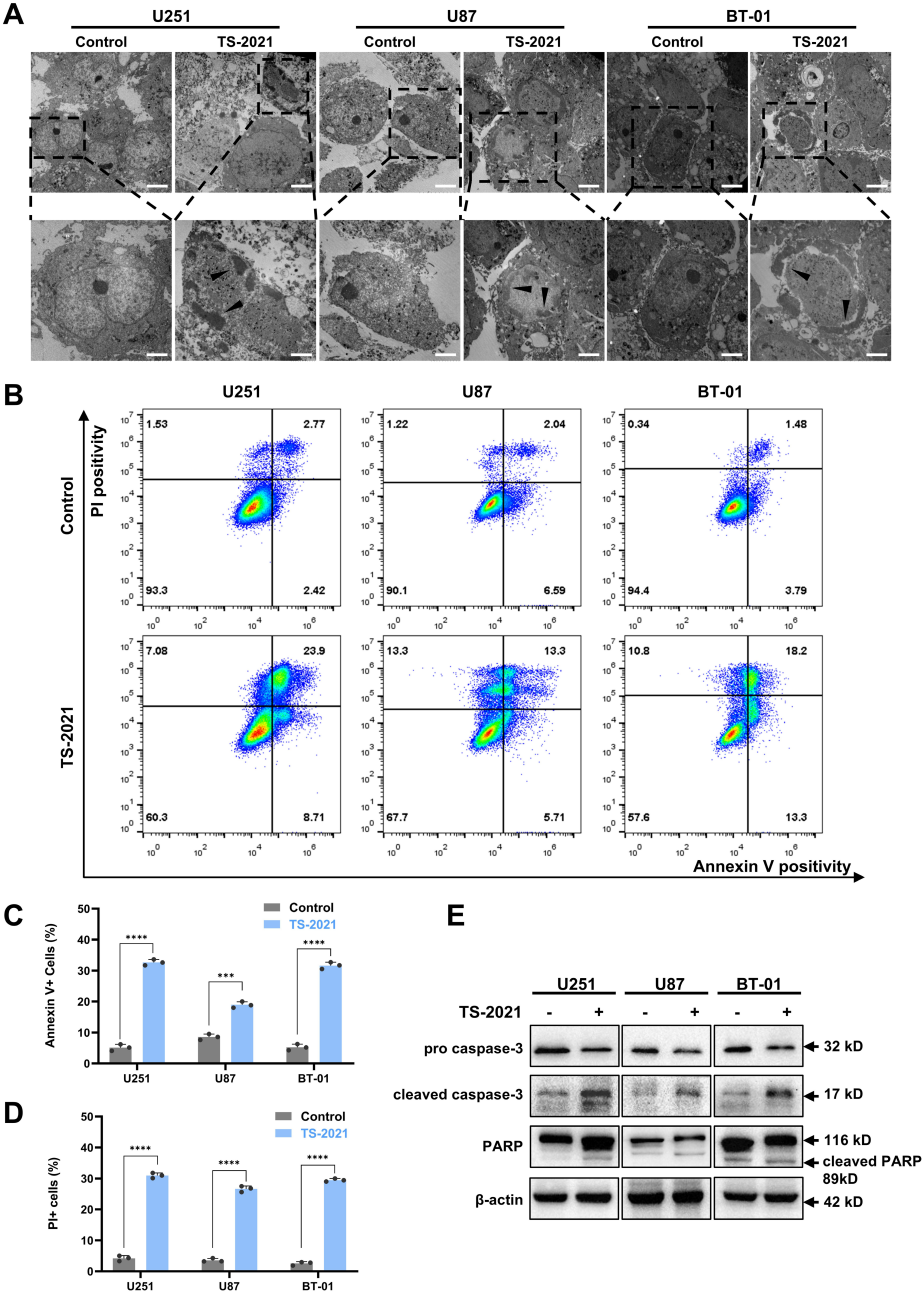
TS‐2021 induced GBM cell death by apoptosis. (A) U251, U87, and BT‐01 cells were infected or not infected (Control) with TS‐2021 (MOI = 300) for 72 h. Typical cell (upper) and mitochondrial (below) ultrastructural images of U251, U87, and BT‐01 cells. Scale bars: Cell, 5 μm; chromatin, 2 μm. (B) Cells were treated as described in A. Cells and supernatants were collected and stained with Annexin V. PI was added simultaneously to evaluate cell membrane damage. The right quadrant shows the average percentage of apoptotic cells; the upper quadrant shows the average percentage of PI+ cells. (C) Quantitative data of Annexin V+ cells in B. (D) Quantitative data of PI+ cells in B. (E) U251, U87, and BT‐01 cells were infected or not infected with TS‐2021 (MOI = 300) for 72 h, and expressions of apoptosis‐maker proteins were detected by western blotting. β‐actin was used as a loading control. Data were analyzed by independent‐samples *t*‐test or one‐way ANOVA with tukey's multiple comparisons test. ***p < 0.001, ****p < 0.0001.

To investigate the cell death pathway caused by TS‐2021, we examined cellular morphological changes 72 h after infection using electron microscopy. We observed cytoplasmic condensation, reduced cell volume, and gradual aggregation of chromosomes into crescent‐shaped attachments around the nuclear membrane with enhanced alkalinity (Figure 1A). These morphological changes suggested that infection by TS‐2021 led to cell apoptosis.

To further illustrate that TS‐2021 infection can induce cell apoptosis, we conducted Annexin V and PI assays to detect phosphatidylserine exposure and cell membrane permeability, respectively (Figure 1B–D), showed increased Annexin V and PI staining in the three GBM cell lines. Our results further confirmed that TS‐2021 induces cell apoptosis.

Afterward, we used western blotting to analyze the expression of apoptosis‐related proteins. We found that TS‐2021 activated caspase‐3, increasing cleaved caspase‐3 and cleaved PARP expression (Figure 1E).

In summary, we observed that TS‐2021 induced apoptosis in GBM cell lines.

### 3.2 TS‐2021 Infection Induced Cell Cycle Arrest, DNA Damage, and PARP Activation

**FIGURE 2:**
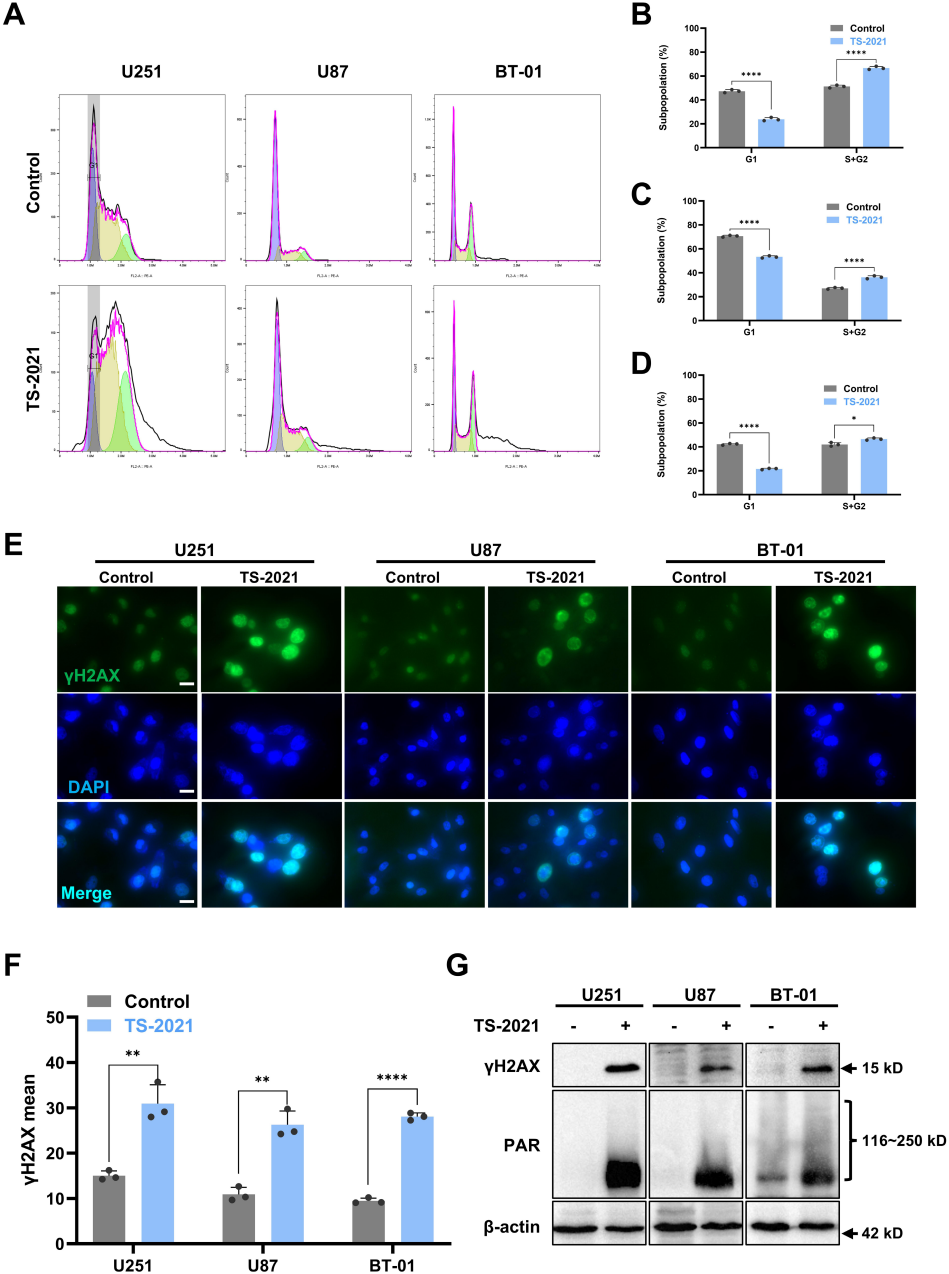
TS‐2021 induced cell cycle arrest, accumulation of γH2AX, and PARP activation. (A) U251, U87, and BT‐01 cells were infected or not infected (Control) with TS‐2021 (MOI = 300) for 72 h. Cell cycle profiles of the cells were detected by flow cytometry. (B) The statistical analysis diagrams of U251 cells in A. (C) The statistical analysis diagrams of U87 cells in A. (D) The statistical analysis diagrams of BT‐01 cells in A. (E) Typical immunofluorescence images of DAPI‐stained cell nuclei and the corresponding distribution of γH2AX labeled with green fluorescence in GBM cells. Scale bar: 20 μm. (F) Quantitative analysis of the mean fluorescence intensity of γH2AX in E. (G) Cells were treated as described in A. γH2AX and PAR expression were analyzed by western blotting. β‐actin was used as a loading control. Data were analyzed by independent‐samples *t*‐test or one‐way ANOVA with Tukey's multiple comparisons test. *p < 0.05, **p < 0.01, ****p < 0.0001.

To determine the mechanism of TS‐2021‐induced cell apoptosis, we analyzed the changes in the cell cycle after infection with TS‐2021. As shown in Figure 2A–D, TS‐2021 induced the cell cycle arrest, indicating increased DNA synthesis, which can result in DNA damage [23]. Therefore, we examined the expression of γH2AX, a highly sensitive indicator of early response to DNA damage in cells [24]. As shown in Figure 2E,F, TS‐2021 increased γH2AX foci formation in cells, resulting in an elevated proportion of γH2AX‐expressing cells. Immunofluorescence staining also confirmed the upregulation of γH2AX expression (Figure 2G). The results above indicated that TS‐2021 infection causes DNA damage, and we subsequently tested the activation of DDR. By western blotting, we found an increase in poly‐ADP‐ribosylation (PARylation) proteins, a marker of PARP activity, indicating significant PARP activation in the three GBM cell lines after infection with TS‐2021 (Figure 2G), suggesting a strong DDR in these cells. Our results showed that TS‐2021 infection can lead to DNA damage and trigger DDR.

### 3.3 TS‐2021 Plus Olaparib Increased Cell Apoptosis and DNA Damage and Decreased PARP Expression

**FIGURE 3:**
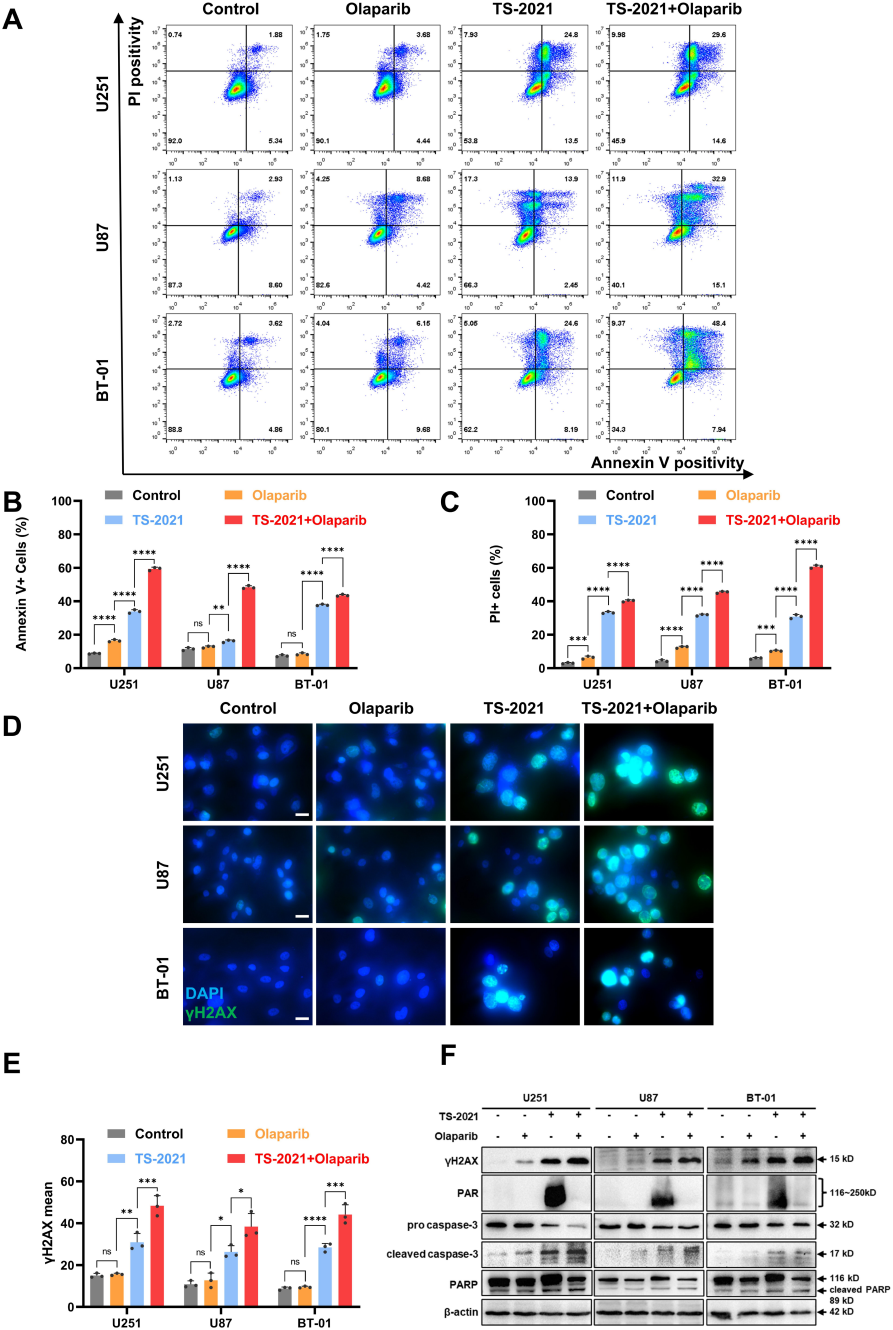
Olaparib increased TS‐2021‐induced cell apoptosis and accumulation of γH2AX. (A) U251, U87, and BT‐01 cells were treated with olaparib (1 μM) and infected with TS‐2021 (MOI = 300) for 72 h. Typical flow‐cytometry analysis image of apoptotic cells ratio in U251, U87, and BT‐01 cells. (B) Quantitative data of Annexin V+ cells in A. (C) Quantitative data of PI+ cells in A. (D) Typical immunofluorescence images of DAPI‐stained cell nuclei and the corresponding distribution of γH2AX in cells treated as described in A. Scale bar: 20 μm. (E) Quantitative analysis of the mean fluorescence intensity of γH2AX in D. (F) Cells were treated as described in A. The expression of γH2AX, PAR, and apoptosis‐maker proteins were detected by western blotting. β‐actin was used as a loading control. PAR, Parylated proteins. Data were analyzed by independent‐samples *t*‐test or one‐way ANOVA with Tukey's multiple comparisons test. *p < 0.05, **p < 0.01, ***p < 0.001, ****p < 0.0001.

As the infection of TS‐2021 caused significant PARP activation and DDR, we explored whether PARP inhibitors can increase viral oncolytic activity. To verify this hypothesis, we administered a conventional PARP inhibitor, olaparib [25]. Flow cytometry showed that combination therapy with olaparib and TS‐2021 increased the proportions of PI‐expressing and annexin V+ cells compared to TS‐2021 monotherapy (Figure 3A–C). Western blotting also corroborated these findings. Compared to TS‐2021 monotherapy, combination therapy increased cleaved caspase‐3 and cleaved PARP expression (Figure 3F). Our findings suggested that combination therapy induces stronger apoptosis than monotherapy.

Furthermore, we examined the PARP activation and DNA damage. Western blotting demonstrated that olaparib effectively inhibited PARP activation by TS‐2021 (Figure 3F). Theoretically, inhibition of PARP activation will increase DNA damage. Afterward, we investigated the DNA damage situation. Immunofluorescence staining showed increased γH2AX accumulation after the combination therapy compared to that after TS‐2021 monotherapy (Figure 3D,E), confirming the western blotting results (Figure 3F). We also observed that olaparib monotherapy had no impact on cell apoptosis, DNA damage, and PARP activation.

The above results confirmed that combination therapy inhibits PARP activation, further enhancing the induction of DNA damage and cell apoptosis.

### 3.4 Olaparib Promoted TS‐2021/Ad5 KT‐E1A‐IL15 and AdGFP/Ad5 KT‐E1A‐GFP Replication and Release in Cells

**FIGURE 4:**
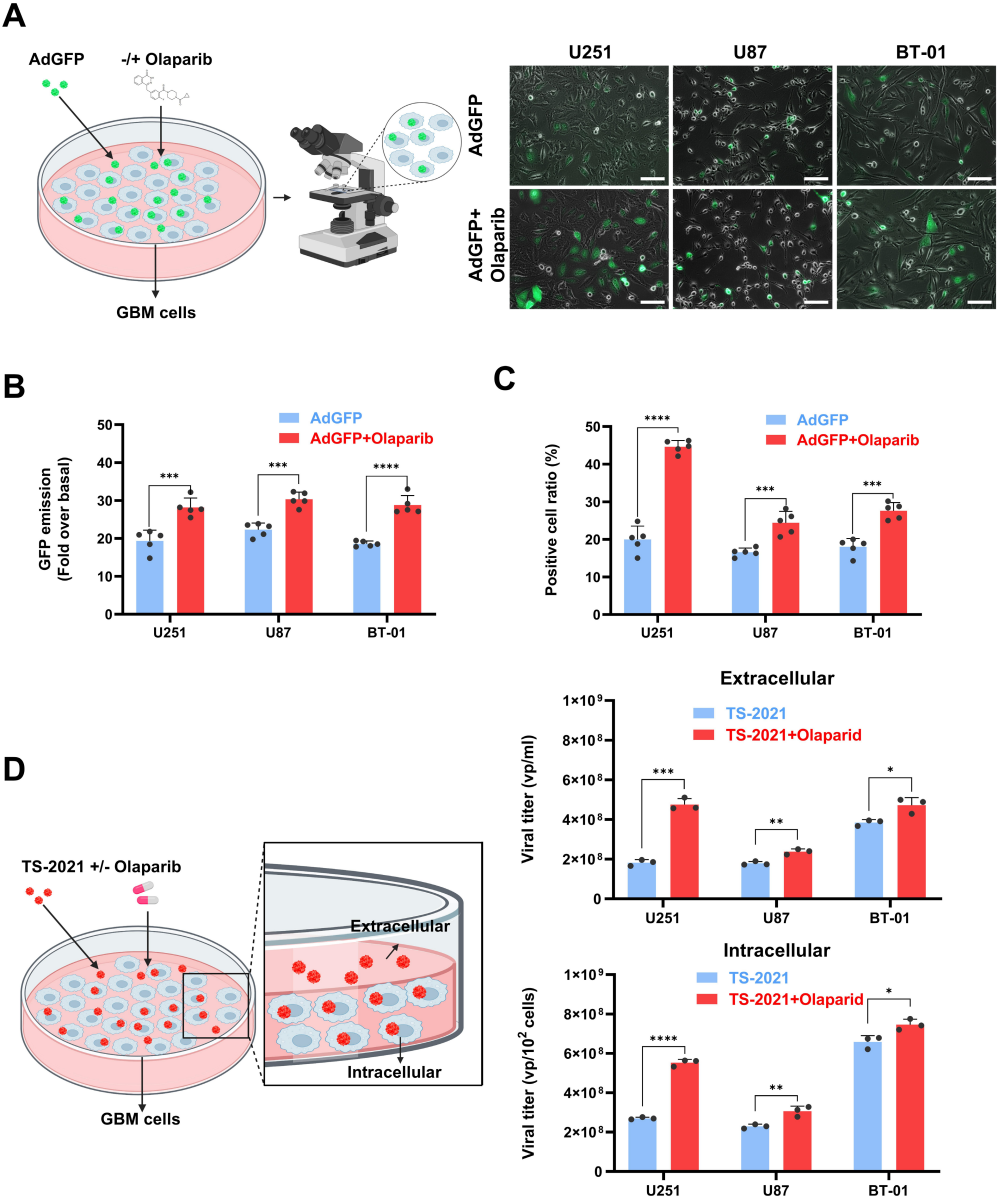
AdGFP/TS‐2021 replication and release in GBM cells were affected by olaparib. (A) U251, U87, and BT‐01 cells were infected with AdGFP (MOI = 300) and treated with or without olaparib (1 μM) for 48 h. GFP intensity was observed under a fluorescence microscope. Scale bars: 100 μm. (B) Quantitative analysis of the GFP emission in A. (C) Quantitative analysis of the GFP‐positive cell ratio in A. (D) U251, U87, and BT‐01 cells were infected with TS‐2021 (MOI = 300) and treated with or without olaparib (1 μM) for 48 h. Adherent cells and supernatant were collected separately. Then, virus DNA was extracted to quantify intracellular and extracellular virus titer through qPCR. Data were analyzed by independent‐samples *t*‐test or one‐way ANOVA with Tukey's multiple comparisons test. *p < 0.05, **p < 0.01, ***p < 0.001, ****p < 0.0001.

To investigate the mechanism of combination therapy, we first studied the impact of olaparib on virus entry and replication. We used AdGFP to infect GBM cells, enabling better visualization of virus replication. Fluorescence microscopy of GBM cells infected with AdGFP after olaparib treatment manifested increased intracellular fluorescence intensity compared to the control group (Figure 4A). Figure 4B,C shows increased GFP emission and proportion of GFP‐positive cells for all three cell lines. The data indicated that olaparib can enhance the replication of AdGFP into GBM cells. Subsequently, we further investigated the effect of olaparib on the replication and release of TS‐2021 in GBM cells. Studies have shown that qPCR can approximately quantify adenovirus titers by designing primers for the sequence of adenovirus E1A region [26, 27]. Furthermore, we evaluated the intracellular and extracellular titers of TS‐2021 in combination with or without olaparib by qPCR. As shown in Figure 4D, after olaparib treatment, both intracellular and extracellular viral titers significantly increased. Our results indicated that olaparib can increase the titer of TS‐2021 in cells and supernatant. In summary, olaparib could potentially play a vital role in promoting TS‐2021 infection and replication processes.

### 3.5 Olaparib Plus TS‐2021 Induced Tumor Cell Apoptosis by Increasing p66shc Phosphorylation

**FIGURE 5:**
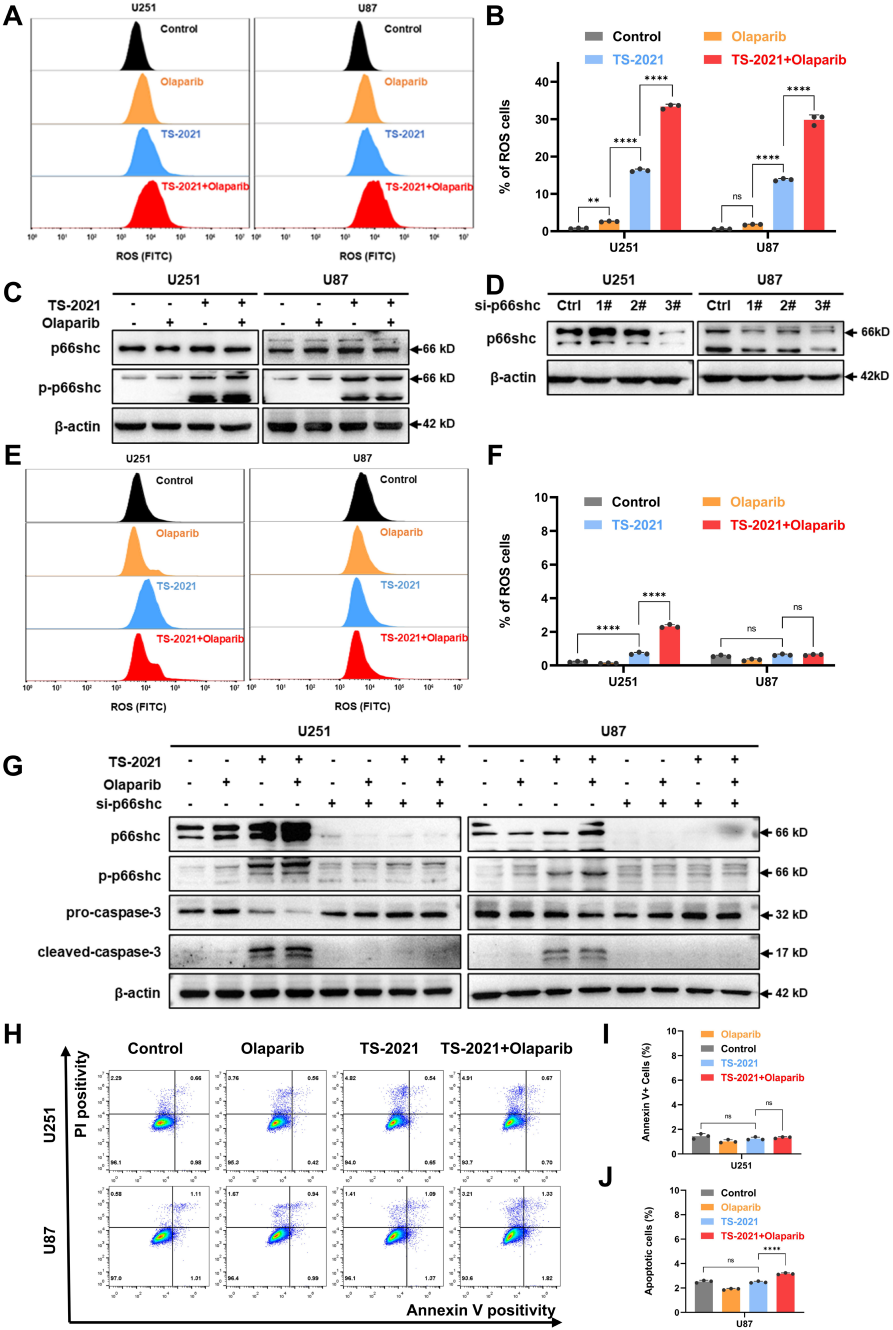
Combination therapy induced phosphorylation of p66shc to increase ROS production, leading to cell apoptosis. (A) U251 and U87 cells were treated with olaparib (1 μM) and infected with TS‐2021 (MOI = 300) for 72 h. Then, the cells were incubated with DCFH‐DA for 1 h in a 37°C cell culture incubator. Cellular ROS level was detected by flow cytometry. (B) Quantitative analysis of the ROS‐positive cell ratio in A. (C) Expression of p66shc and its phosphorylated protein in U251 and U87 cells with the same treatment above. β‐Actin was used as a loading control. (D) The efficacy of p66shc silencing was assessed by western blotting. β‐actin was used as a loading control. (E) U251 and U87 cells were pretreated with siRNA3# (20 μM) for 48 h, then the cells were treated as described in A. Cellular ROS level after p66shc silencing was detected by flow cytometry. (F) Quantitative analysis of the ROS‐positive cell ratio in E. (G) U251 and U87 cells with or without p66shc silencing were treated with olaparib (1 μM) and infected with TS‐2021 (MOI = 300) for 72 h. The expression of p66shc, phosphorylated protein and apoptosis‐related proteins. β‐actin was used as a loading control. (H) U251 and U87 cells with p66shc silencing were treated with olaparib (1 μM) and infected with TS‐2021 (MOI = 300) for 72 h. The ratio of apoptotic cells is determined by flow cytometry. (I) Quantitative data of U251 in H. (J) Quantitative data of U87 in H. Data were analyzed by independent‐samples *t*‐test or one‐way ANOVA with tukey's multiple comparisons test. **p < 0.01, ****p < 0.0001.

Next, we investigated the mechanisms of how combination therapy increases cell apoptosis. ROS analysis using the fluorescent probe DCFH‐DA showed that the combination therapy induced higher ROS production than TS‐2021 and olaparib monotherapies (Figure 5A,B).

P66shc is the only oxidoreductase involved in mitochondrial ROS generation [28]. Western blotting demonstrated that olaparib plus TS‐2021 promoted p66shc phosphorylation (Figure 5C). Furthermore, to demonstrate the pivotal role of p66shc in promoting ROS production and cell apoptosis in combination therapy, we opted to silence p66shc using three types of siRNA and assessed its effectiveness. Western blotting identified siRNA3# as the most efficient p66shc‐targeting siRNA (Figure 5D).

After silencing p66shc, we performed ROS and cell apoptosis detection again. DCFH‐DA staining showed that p66shc silencing decreased ROS levels in the combination and TS‐2021 groups (Figure 5E,F). Western blotting indicated significant inhibition of p66shc phosphorylation and cleaved caspase‐3 expression (Figure 5G). The proportion of apoptotic cells decreased by p66shc silencing in both the combination and TS‐2021 groups (Figure 5H–J). Our findings suggested that p66shc promotes ROS production and is essential for TS‐2021 monotherapy‐ and combination therapy‐induced apoptosis.

### 3.6 Olaparib Enhanced the Oncolytic Activity of TS‐2021 In Vivo

**FIGURE 6:**
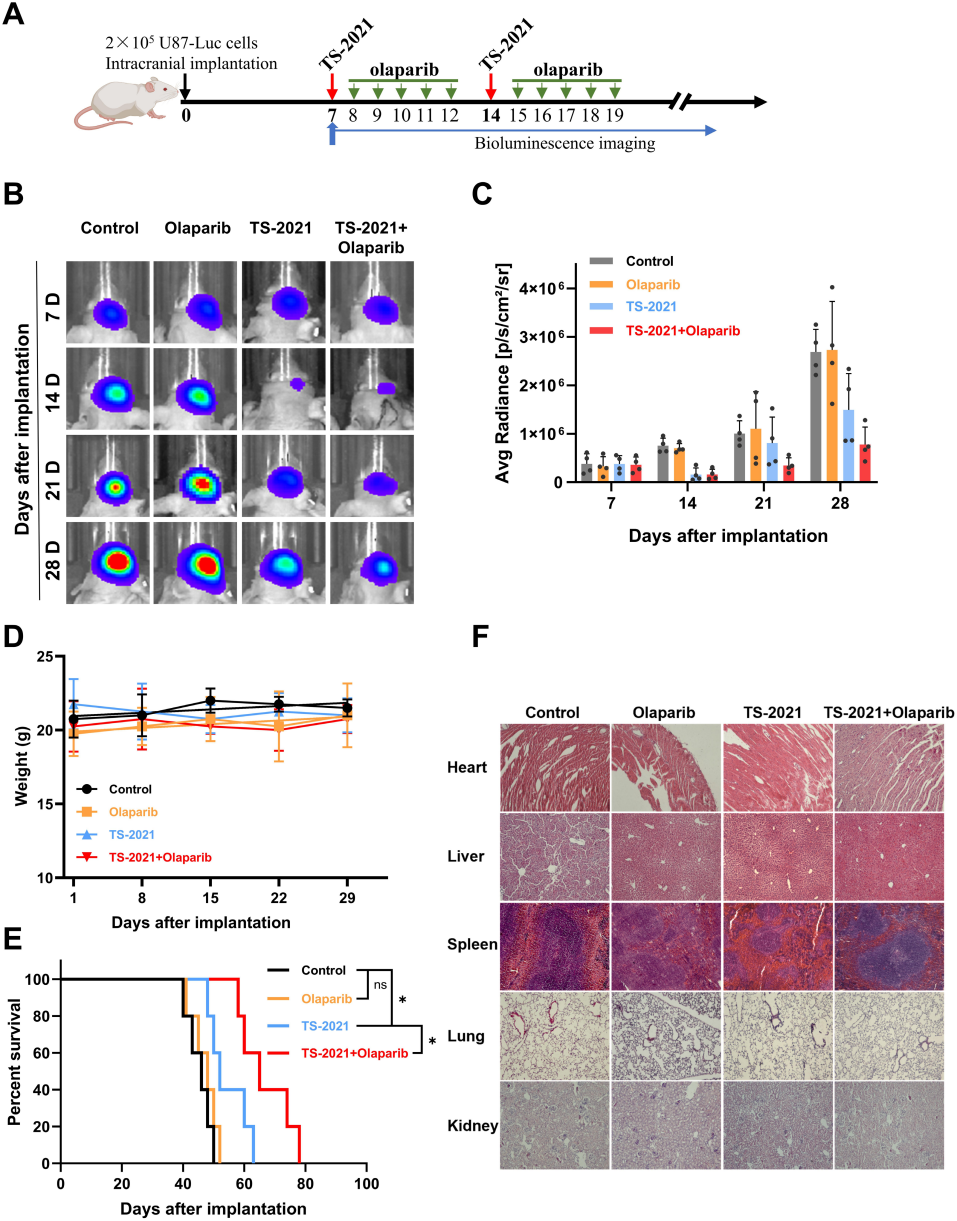
Combination therapy suppressed GBM growth and prolonged survival time in mice. (A). Schematic diagram of animals in vivo experiment. (B) BALB/c nude mouse model of intracranial GBM was established by in situ injection of U87‐Luc cells. Mice were administrated with TS‐2021 by intracranial injection 7 and 14 days after tumor implantation; olaparib was orally administrated via gavage for five consecutive days after injecting TS‐2021. IVIS images were captured 7, 14, 21, and 28 days after tumor implantation. (C) Associated ROI quantification of tumors in B. (D) The weights of mice in each group were recorded at 1, 8, 15, 22, and 29 days after implantation. (E) The survival curve of all mice was monitored until death. Data are presented as mean ± SD. ns *p* > 0.05, *p < 0.05. (F) Typical hematoxylin and eosin staining images of heart, liver, spleen, lungs, and kidney tissue in each group of mice. Scale bars: 100 μm.

To evaluate the antitumor effect of olaparib plus TS‐2021, we established a patient‐derived xenograft model of GBM in mice using U87‐Luc cells. Mice were injected with TS‐2021 in situ and orally administered olaparib via gavage or treated with olaparib plus TS‐2021. IVIS images and ROI quantification (Figure 6B,C) showed that olaparib monotherapy did not significantly affect tumor growth, while TS‐2021 plus olaparib significantly inhibited tumor growth. There were no significant differences in body weight between the four groups (Figure 6D). Combination (median survival = 65 days) therapy significantly improved survival compared to TS‐2021 (median survival = 52 days) and olaparib (median survival = 48 days) monotherapies (Figure 6E). Morphological analyses of the heart, liver, spleen, lung, and kidney showed no obvious toxicity (Figure 6F).

## 4 Discussion

Patients with GBM, one of the deadliest human malignancies, have a median overall survival period of approximately 15 months [29]. Oncolytic virotherapy is among the most promising GBM treatment strategies. To further improve oncolytic virotherapy, it is crucial to overcome the host antiviral response. In this study, we demonstrated the synergistic antitumor effect of the oAd TS‐2021 and the PARP inhibitor olaparib in combination therapy, providing a new strategy to improve the efficacy of oncolytic virotherapy for GBM.

DDR induced by oncolytic viruses, such as bovine herpesvirus1 [30] and canine parvovirus [31], plays an important role in host antiviral response [32]. PARP inhibitors are molecularly targeted drugs that inhibit DDR, leading to the sustained existence of DNA single‐strand breaks (SSBs) that eventually transform into DNA double‐strand breaks (DSBs) [15]. In HR‐deficient cancers, the accumulation of DSBs leads to cell death, which aligns with homozygous lethality [33]. However, in HR‐proficient cancers, DSBs are repaired [34]. GBM, an HR‐proficient tumor, is difficult to treat with PARP inhibitor monotherapy [16, 17]. However, PARP inhibitors combined with other therapies can inhibit HR‐proficient tumor growth [35]. For instance, PARP inhibitors combined with chemotherapy (TMZ [36], platinum salts [37], gemcitabine [38], etc.) or targeted drugs (EGFR inhibitors [39], HSP90 inhibitors [40], etc.) exert therapeutic effects on malignant tumors. The oAd dl922‐947 causes DNA damage and exhibits a synergistic antitumor effect when combined with olaparib [41, 42]. In our study, combination therapy with TS‐2021 and olaparib exhibited synergistic antitumor effects, inducing cell cycle arrest and cell apoptosis. Meanwhile, we observed for the first time that olaparib can enhance TS‐2021 infection in GBM cells, increasing both intracellular and extracellular viral titers.

PARP activation initiates DDR via PARylation [43, 44], and cleavage of PARP indicates apoptosis and caspase activation [45]. In our study, we initially observed that TS‐2021 activated PARP. Subsequently, olaparib and TS‐2021 synergistically increased γH2AX expression and DNA damage, which are related to the inhibition of PARP activity. Moreover, olaparib combined with TS‐2021 promoted apoptosis, as evident from caspase‐3 activation and PARP cleavage. During TS‐2021 infection, PARP inhibition increased caspase‐dependent apoptosis and DNA damage accumulation. In vivo studies further confirmed the potential therapeutic effect of olaparib combined with TS‐2021. First, we analyzed the effect of the combination therapy on GBM xenograft tumors by injecting U87‐Luc cells in nude mice. Olaparib and TS‐2021 combination therapy significantly reduced tumor volume and prolonged mouse survival compared to monotherapies with these agents. Subsequently, we validated the nontoxicity of the combination therapy by monitoring mouse body weight, which remained stable throughout the study. In addition, there were no changes in heart, liver, spleen, lung, and kidney functions. Combination therapy with TS‐2021 and olaparib demonstrated enhanced antitumor effects in vitro and in vivo.

Increased ROS production is observed in cells with various viral infections and is vital in inducing DNA damage and cell death. ROS typically induces cell death by activating caspase‐induced apoptosis [46]. Subsequently, apoptotic bodies are formed through extrinsic and intrinsic pathways and engulfed by phagocytic cells [47, 48]. P66shc, a member of the ShcA protein family, is a crucial oxidoreductase that participates in mitochondrial ROS production. Phosphorylation of its Ser36 residue under oxidative stress leads to apoptosis [49]. Oncolytic viruses can act as an exogenous factor inducing cellular oxidative stress to inhibit tumor growth [50]. Our results showed that the combination of olaparib and TS‐2021 increased Ser 36 phosphorylation in p66shc and enhanced ROS production compared to monotherapy with either agent. Furthermore, p66shc silencing with siRNA significantly reduced ROS production in GBM cells. Meanwhile, the combination therapy‐induced apoptosis was offset by p66shc silencing. This study demonstrated for the first time that combination therapy with TS‐2021 and olaparib synergistically promoted tumor cell killing by enhancing p66shc‐dependent cell apoptosis. Nonetheless, this study has certain limitations. Although we explored a potential mechanism of action for combination therapy with olaparib and TS‐2021, it remains unclear how olaparib promotes virus replication. Further investigations are required to uncover additional targets for improved oncolytic virotherapy.

**FIGURE 7:**
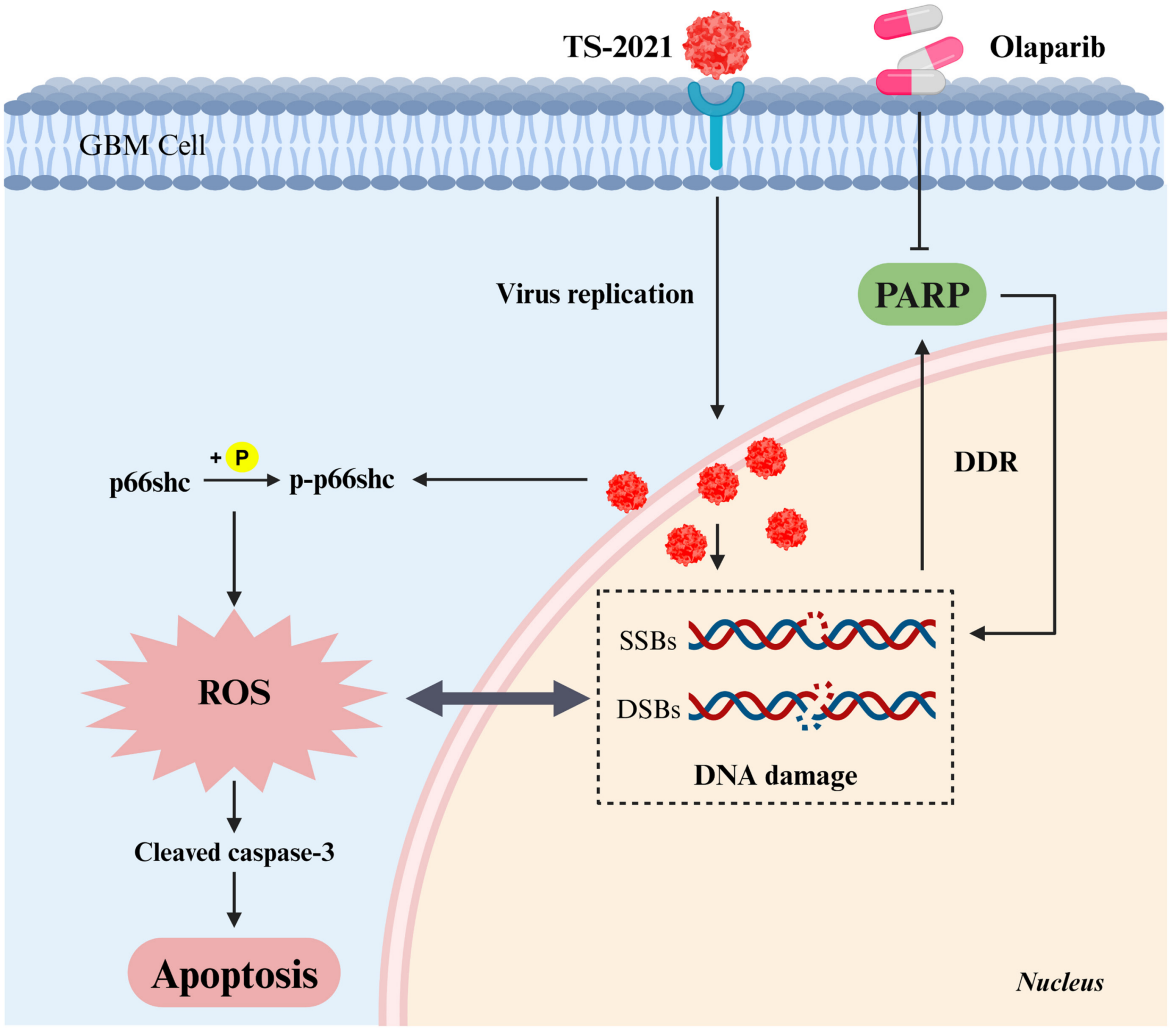
Schematic diagram of TS‐2021 and Olaparib synergistically enhancing anti‐tumor efficacy. TS‐2021 and olaparib synergistically promote the distribution and replication of TS‐2021 in GBM cells, synergistically reduce the activation of PARP, enhance the DNA damage of GBM cells, and up‐regulate the apoptosis of tumor cells induced by p66shc phosphorylation. The above diagram was created by BioRender.com.

In summary, our study showed that combination therapy with olaparib and TS‐2021 was effective against human GBM in vivo and in vitro. Furthermore, we demonstrated that the efficacy of this combination therapy relied on DNA damage and p66shc‐induced cell apoptosis (Figure 7). Overall, we successfully applied olaparib to HR‐proficient GBM by combining it with TS‐2021, offering a novel approach to treating GBM.

## Author Contributions

**Yida Liu:** conceptualization, methodology, investigation, validation, writing – original draft. **Sheng Fang:** methodology, formal analysis, writing – review and editing. **Peiwen Wang:** validation, investigation. **Junwen Zhang:** methodology, supervision. **Fusheng Liu:** resources, supervision, writing – review and editing, project administration, funding acquisition. All authors have read and approved the final manuscript.

## Ethics Statement

The Experimental Animal Ethics Committee of the Beijing Neurosurgery Institute approved the use of animals and experimental procedures.

## Conflicts of Interest

The authors declare no conflicts of interest.

## Supporting information


Figure S1

Data S1

## Data Availability

The data that support the findings of this study are available from the corresponding author upon reasonable request.
