# Supplementary material for: Olaparib Enhances the Efficacy of Third‐Generation Oncolytic Adenoviruses Against Glioblastoma by Modulating DNA Damage Response and p66shc‐Induced Apoptosis
Source: CNS Neurosci Ther. 2024 Nov 18;30(11):e70124. doi: 10.1111/cns.70124 (PMC11570871; doi:10.1111/cns.70124)
Supplement: Supplementary file 1 — Figure S1 [file CNS-30-e70124-s002.docx]

**
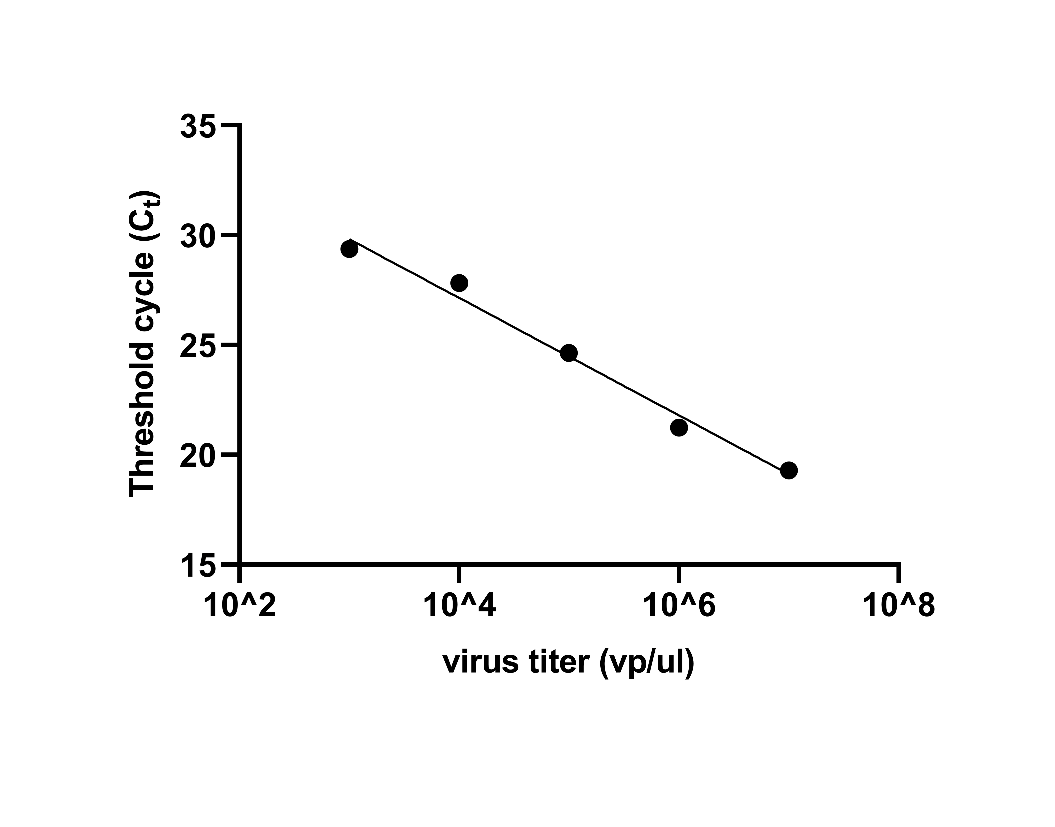
**

**Figure S1.** **The standard reference curve for TS-2021 qPCR assay standards.** A standard curve was generated by plotting the threshold cycle (Ct) against the initial plasmid copy number for each of the standards. The quantitative range of the standard curve was found to be between 1 × 10^2^ and 1 × 10^8^ copies. The resultant line equation can be used to determine the initial copy numbers for unknown samples, based on their Ct values.


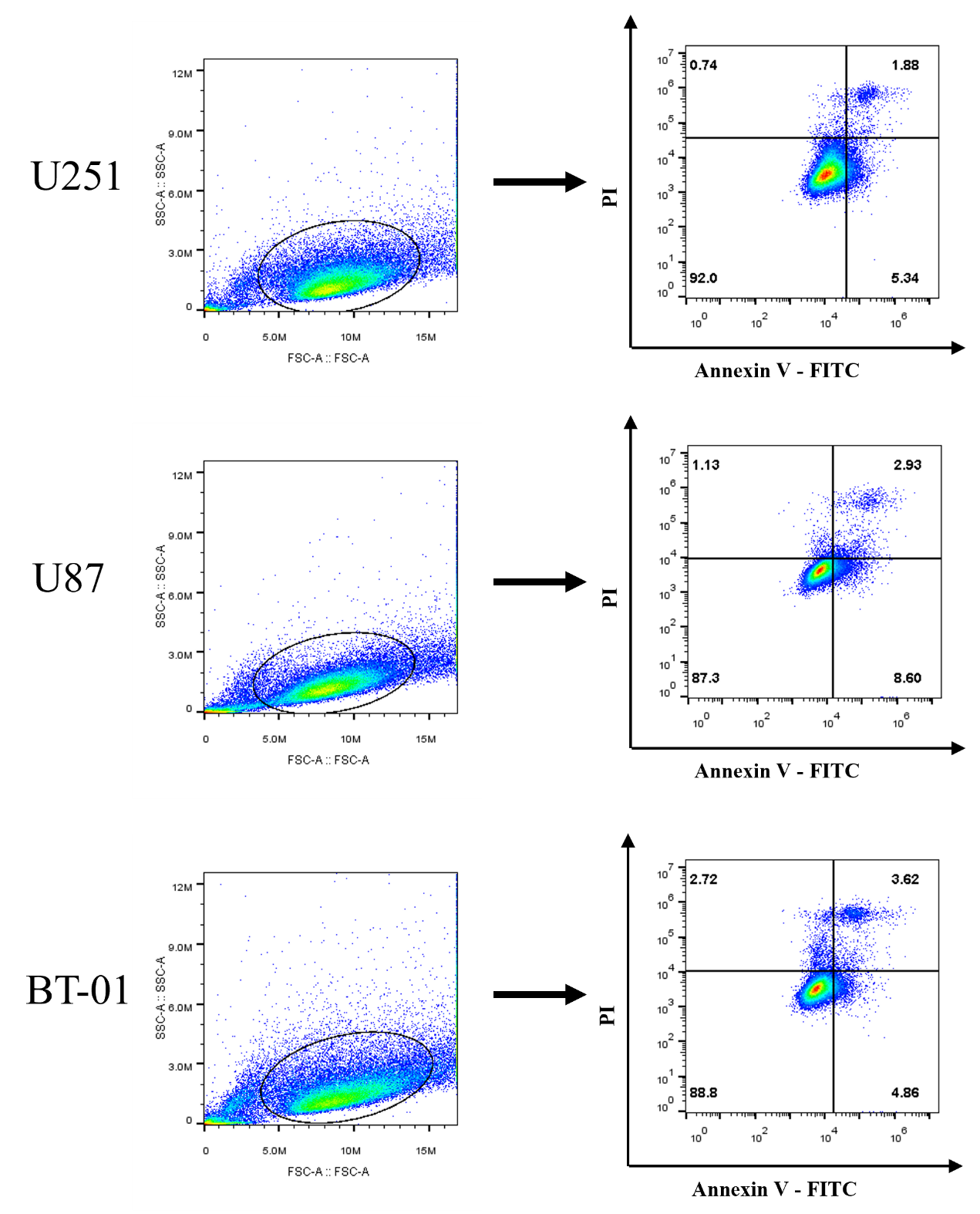


**Figure S2. Gating strategy.**

U87, U251 and BT01 cell colonies were preliminarily gated and using FSC-A and SSC-A values. Then Apoptotic cells were further gated based on PI and FITC fluorescence.
