# Supplementary material for: Olaparib Enhances the Efficacy of Third‐Generation Oncolytic Adenoviruses Against Glioblastoma by Modulating DNA Damage Response and p66shc‐Induced Apoptosis
Source: CNS Neurosci Ther. 2024 Nov 18;30(11):e70124. doi: 10.1111/cns.70124 (PMC11570871; doi:10.1111/cns.70124)

Full unedited gel/blot for Figure 1E

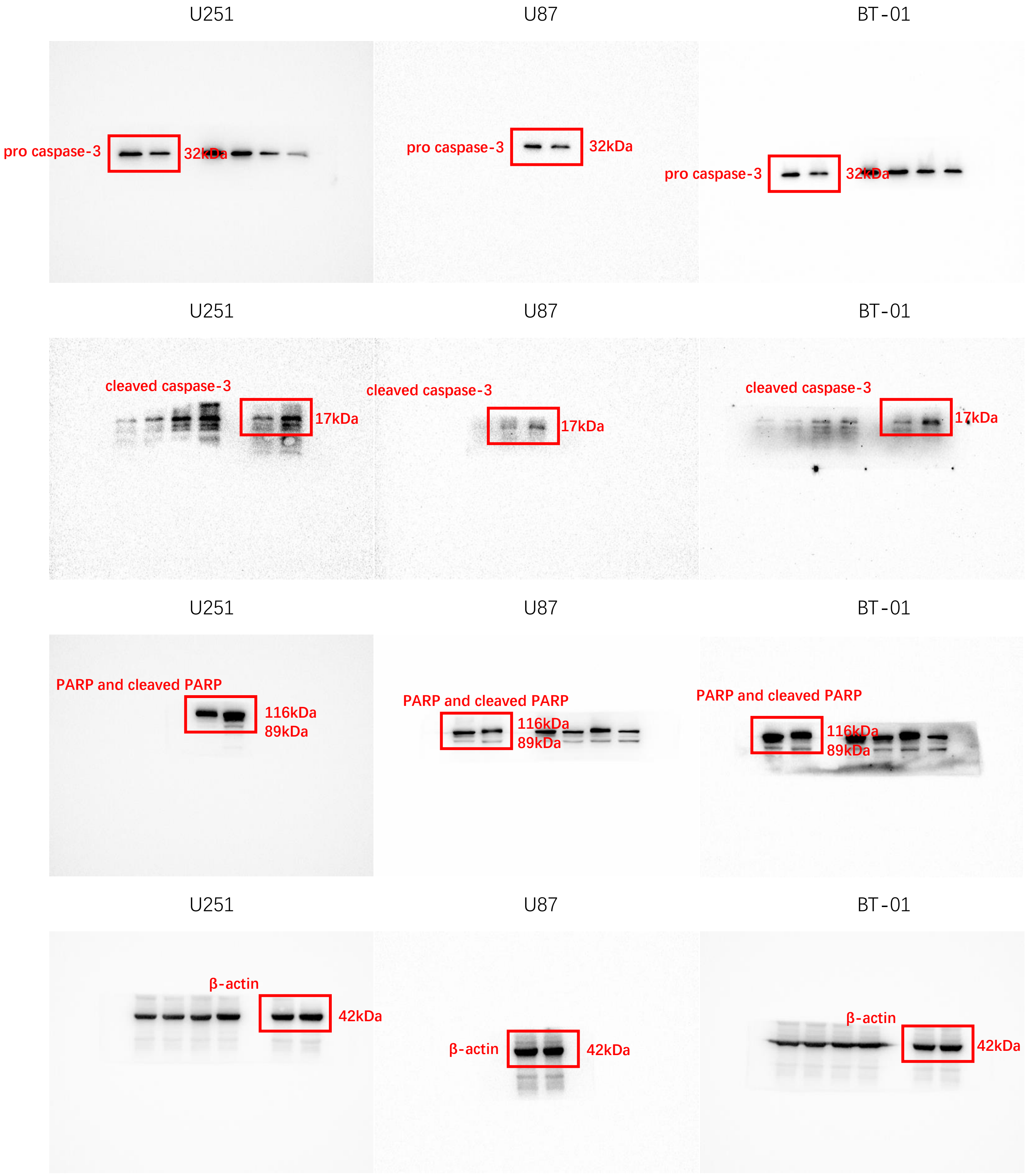

Full unedited gel/blot for Figure 2G

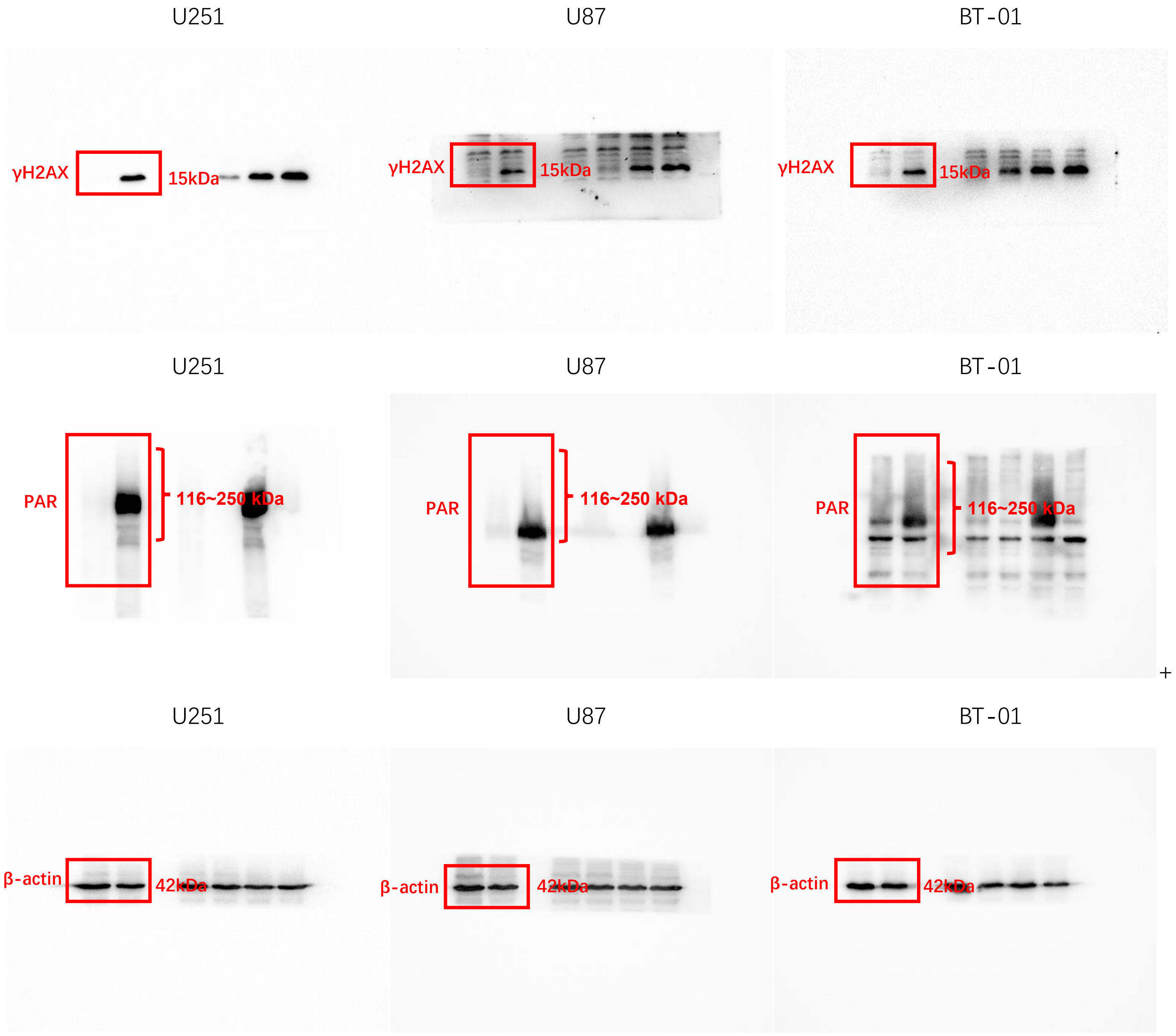

Full unedited gel/blot for Figure 3F

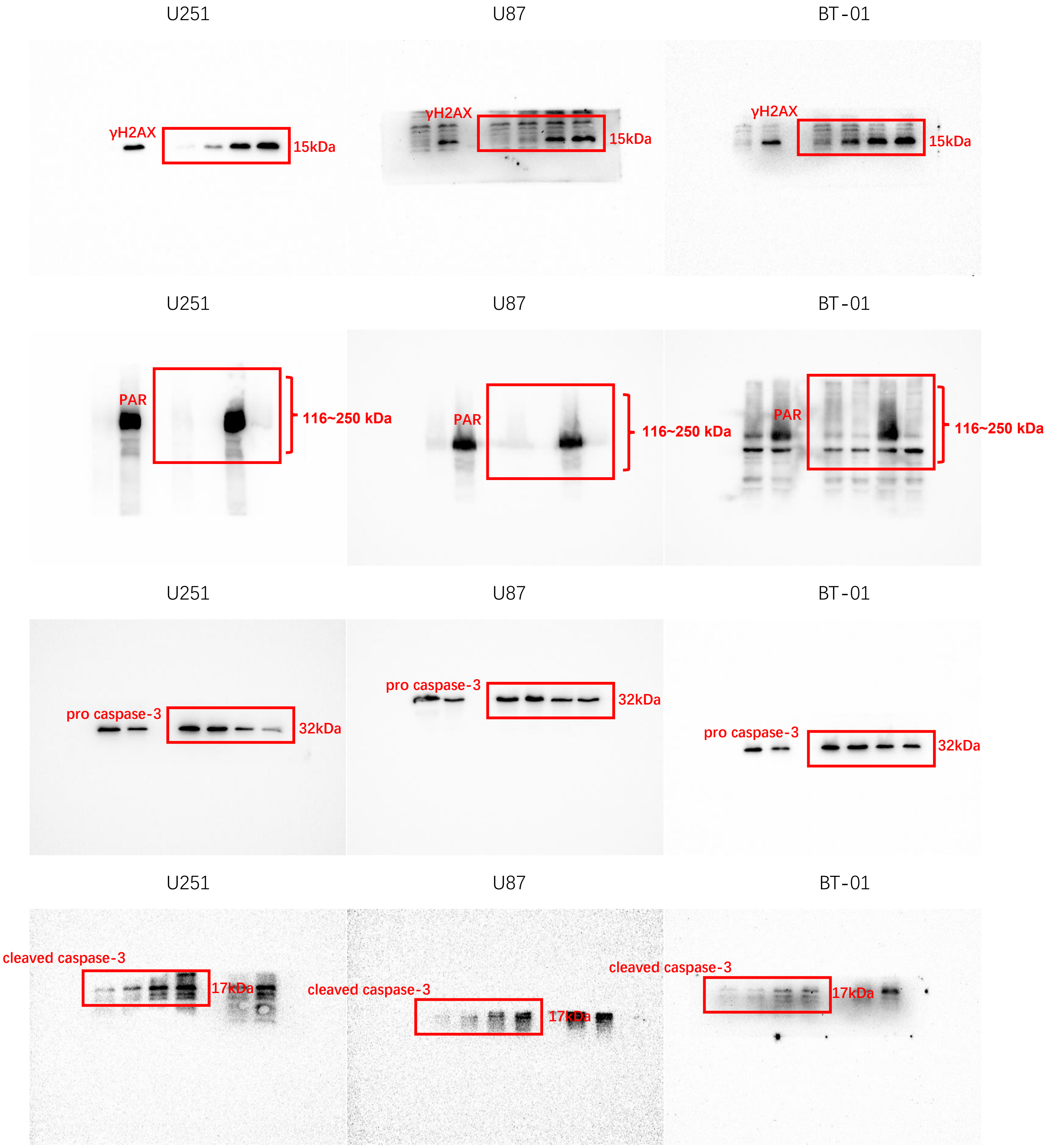

Full unedited gel/blot for Figure 3F

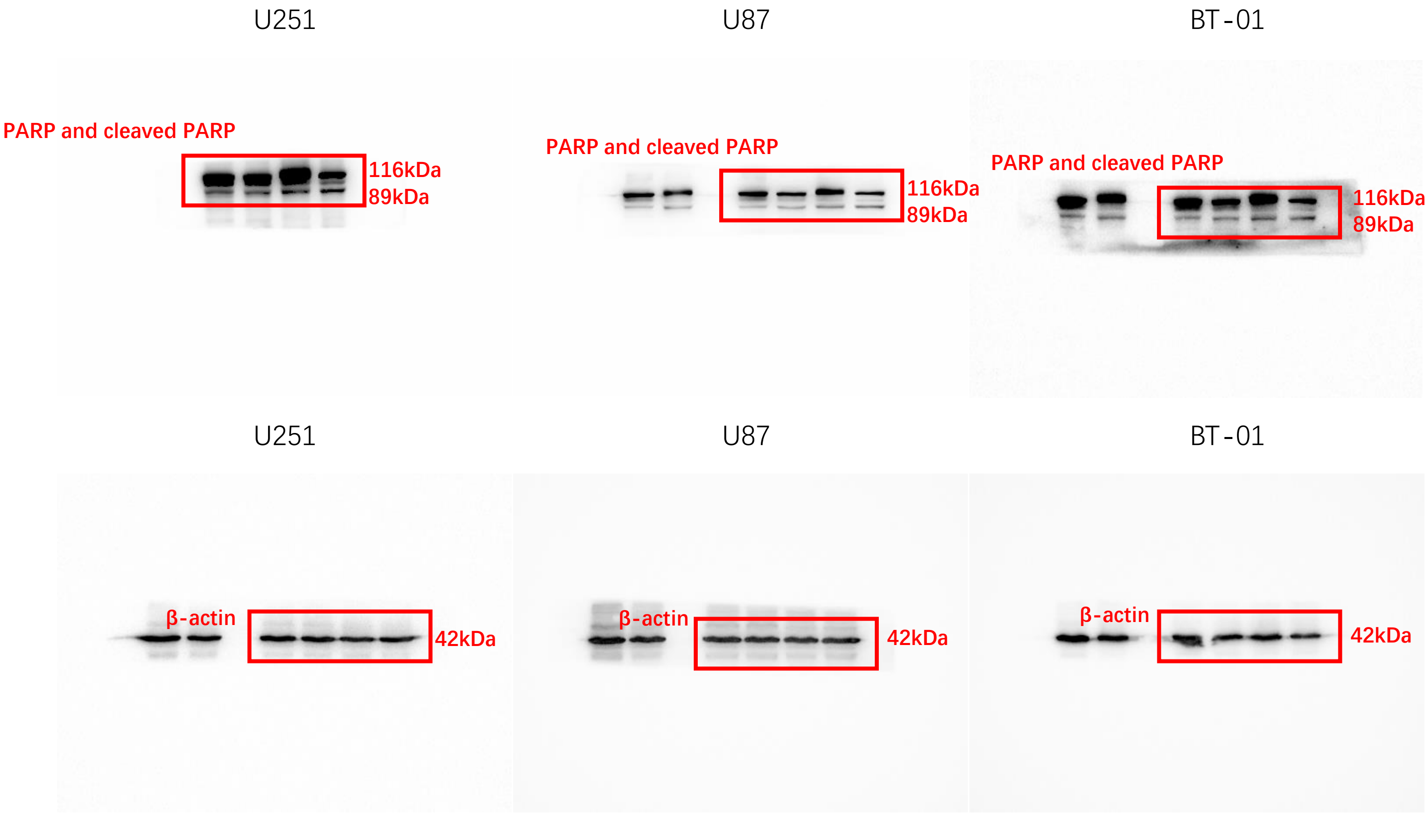

Full unedited gel/blot for Figure 5C

U251

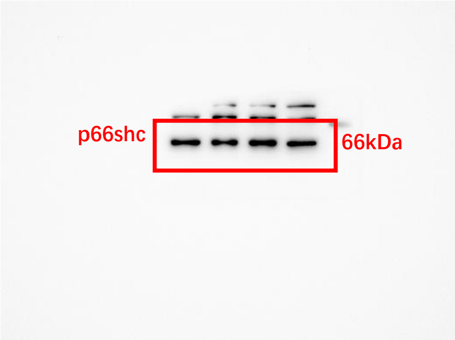

U87

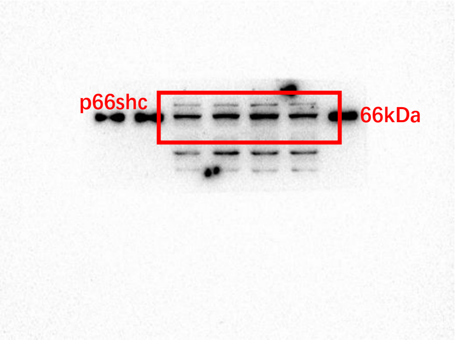

U251

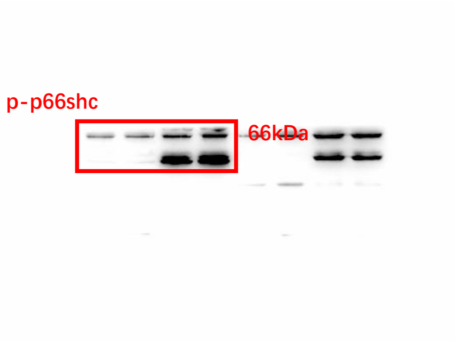

U87

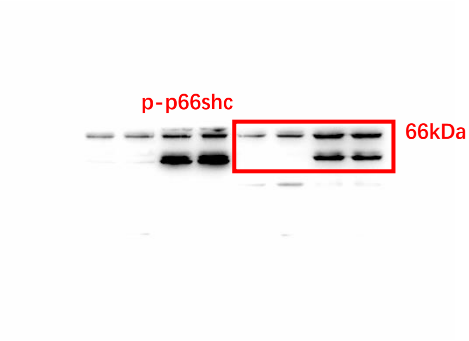

U251

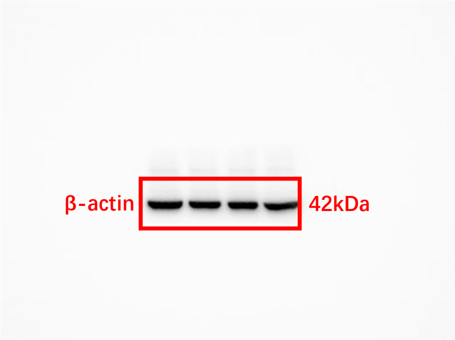

U87

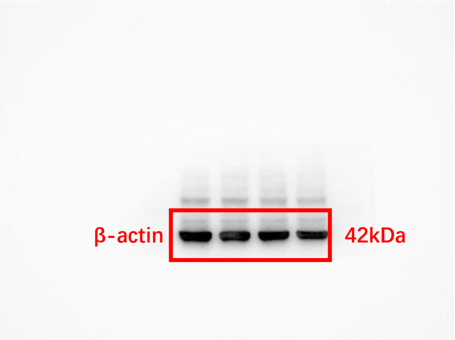

Full unedited gel/blot for Figure 5D

U251

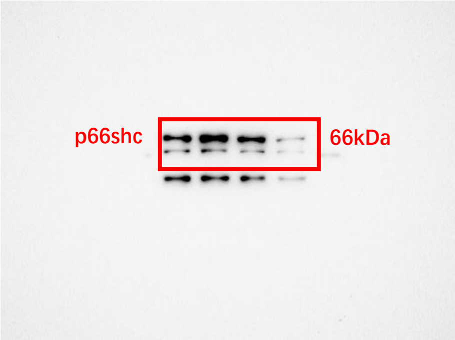

U87

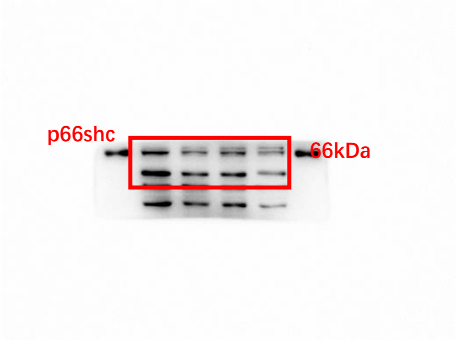

U251

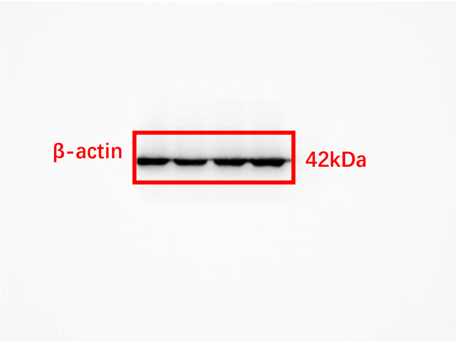

U87

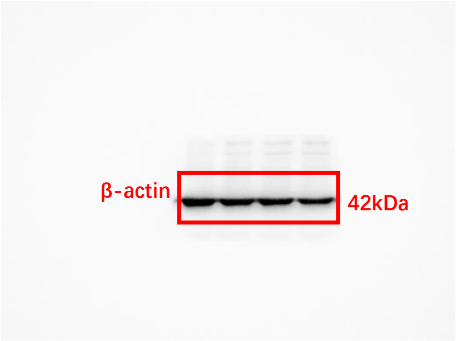

Full unedited gel/blot for Figure 5G

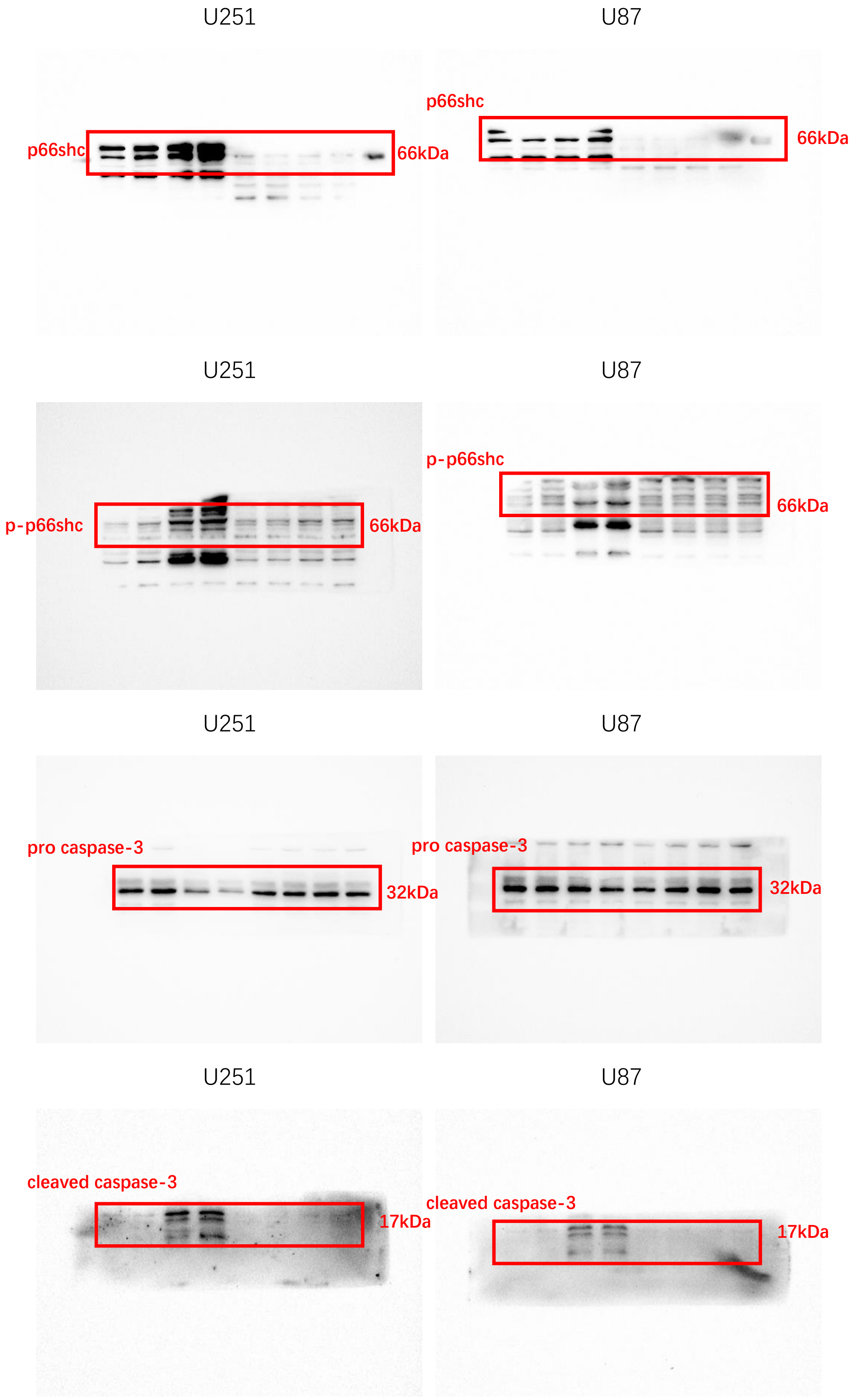

Full unedited gel/blot for Figure 5G

U251

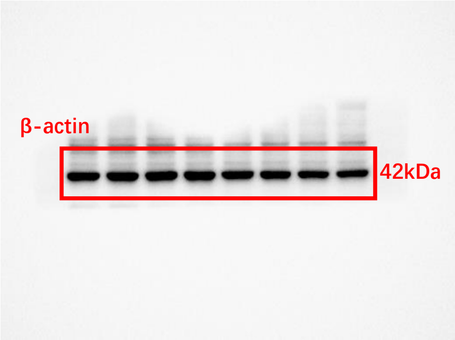

U87

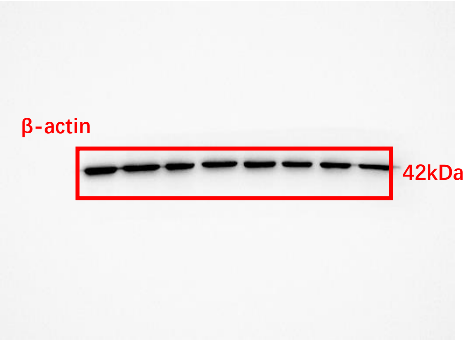

Supplement: Supplementary file 2 — Data S1 [file CNS-30-e70124-s001.pdf]
